# Optimism and survival: health behaviors as a mediator—a ten-year follow-up study of Chinese elderly people

**DOI:** 10.1186/s12889-022-13090-3

**Published:** 2022-04-06

**Authors:** Zhang Yue, Hang Liang, Xigang Qin, Yang Ge, Nan Xiang, Erpeng Liu

**Affiliations:** 1 School of Public Administration, Zhongnan University of Economics and Law, Wuhan, 430073 China; 2 Institute of Income Distribution and Public Finance, Zhongnan University of Economics and Law, Wuhan, 430073 China

**Keywords:** Chinese elderly people, Optimism, Health behaviors, Survival, Mediating effect, Follow-up study

## Abstract

**Background:**

Optimism—the generalized expectation that good things will happen—is a promising health asset. Mounting evidence indicates that there are specific associations between optimism and survival rates. However, for public health purposes, it is critical to consider whether the relationship between optimism and survival holds for older adults as a whole and to explore the role of health behaviors as potential mediators.

**Methods:**

Prospective data were obtained from the Chinese Longitudinal Healthy Longevity Survey (CLHLS). Optimism was measured in 2008, and survival was measured by survival time of the interviewees during the whole observation period from 2008 to 2018. Cox proportional hazard models were employed to evaluate the association between optimism and survival among the elderly. The mediating effect analysis method was used to explore the potential mediating role of health behaviors on the association between optimism and survival.

**Results:**

Compared to less optimistic older adults, optimistic individuals were associated with lower odds of mortality (HR = 0.94, 95% CI = 0.89 − 0.99). Health behaviors are key elements that play a positive role in survival (HR = 0.95, 95% CI = 0.94 − 0.96). Health behaviors played an intermediary role in the relationship between optimism and mortality, and the mediating effect was -0.005.

**Conclusions:**

Optimism and health behaviors were broadly and robustly associated with a lower risk of mortality. Health behaviors mediate the relationship between optimism and mortality. Appropriate intervention should be carried out on optimism and health behaviors among elderly people to improve the likelihood of health in aging.

## Background

Optimism is a psychological trait that can be defined as the general expectation that good things, rather than bad things, will occur in one’s future [1]. Optimistic individuals tend to expect that they will overcome difficulties and encounter favorable outcomes, and they usually enjoy healthier and longer lives than their less optimistic peers [2, 3]. Optimism is considered a core component of one’s psychological well-being that shapes one’s thoughts and feelings and influences one’s behavior patterns in older age. With the rapid pace of increase of the aging population, rising life expectancy represents the progress of medical sciences, but not all the increased years of life are being spent in optimal health, which has attracted people to explore the factors that influence healthy longevity. Recent studies show that psychological assets such as optimism are potential predictors of longer life, based on findings associating higher optimism with a reduced risk of chronic disease among elderly individuals and premature mortality [4–6].

Do optimists live longer than pessimists? Having a positive outlook or being optimistic may pose an independent health benefit to the elderly [7]. Many studies have consistently demonstrated that higher level of optimism is associated with lower mortality [8–10], lower rates of chronic conditions [11, 12], higher rates of recovery from illness [13, 14], and better evaluation of self-rated health and life satisfaction [15]. In contrast, many studies have reported that negative effects (e.g., depression and uselessness) among elderly individuals are linked with higher rates of functional impairment and disability [16, 17], higher risk of cardiovascular disease [2], and poorer functions of cognition [18]. Importantly, optimism is associated with health outcomes above and beyond its role in signaling the absence of poor psychological function [19] and independent of sociodemographic confounders, health conditions, and lifestyle [20]. Studies further suggest that optimistic elderly individuals tend to foster a variety of positive behaviors (e.g., less cigarette smoking, more exercise), which could significantly improve elderly individuals’ quality of life [21–23].

One pathway through which optimism might affect physical and mental well-being is by the maintenance of prudent health behaviors [10, 24, 25]. Associations between optimism and health behaviors in older people might be expected on both theoretical and empirical grounds. In addition to the potential direct effects of optimism on physiological processes, as well as its possible role buffering against the harmful consequences of stress, theorists posit that individual with greater optimism enjoy better health outcomes because they engage in healthier behaviors such as exercising, eating fruits and vegetables, and avoiding cigarette smoking [26, 27]. A recent study suggested that optimism is conducive to the formation of health behaviors, which play a key role in reducing the risk of chronic diseases such as hypertension and stroke [28, 29]. Many studies have illustrated that health behaviors are associated with a reduced risk of mortality [30, 31] and improved physical and psychological wellness [32, 33]. There may be confusion that regular health behaviors might be cultivated at an early age among elderly individuals, and the positive effect between higher level of optimism and higher longevity could be overstated. Studying the association between baseline optimism and health behaviors in old age could reduce this reverse causality.

Although some work has documented associations between optimism and mortality, chronic diseases and health behaviors, a few studies have examined optimism as a predictor of survival among elderly individuals [22, 34], and less attention has been given to whether health behaviors have direct and indirect connections with survival among Chinese individuals [30]. Previous studies compounded health status (e.g., self-rated health and cognitive function) when exploring the association between optimism and survival, and which might ignore the fact that optimism would be affected by health condition [24]. It is unclear whether the findings in developed countries are valid in developing countries, such as in China, where survival of older adults is more selective due to high mortality at younger ages, and research on associations between optimism, health behaviors and survival is rare. In addition, the existing literature on optimism based on a large sample is still rare [22, 35], which might limit the generalizability of the findings of such studies.

This study aimed to examine the association between optimism and survival among older adults in China and explore the potential mediating role of health behaviors. The major contributions of this study included mainly the following points. First, health behaviors were explored as a link between optimism and survival, which has been less analyzed in the previous literature. Using longitudinal data, we search for evidence to explore the mediating role of health behaviors, which can help determine whether a causal association is plausible and thereby whether optimism and health behaviors are viable targets for intervention. Second, the study was based on data from a large sample in China over a ten-year follow-up period. China is the largest developing country with a rapidly aging population, where the intervention measures for optimistic psychology and health behaviors are still not perfect. More general conclusions can be drawn from our study based on the large sample in China, which may also have implications for other countries experiencing population aging.

## Methods

### Data source and study sample

We used data from the Chinese Longitudinal Healthy Longevity Survey (CLHLS). The CLHLS has been collecting a comprehensive data set from elderly individuals since 1998. To date, seven follow-up waves of data were collected in 2000, 2002, 2005, 2008, 2011, 2014 and 2018. The survey randomly selected half of the total number of counties and cities in 22 out of 31 provinces in mainland China, and the 22 provinces where CLHLS was conducted covered approximately 85% of the total population of China [36]. Extensive data were collected in the CLHLS using internationally standardized questionnaires adapted to the Chinese cultural and social context. The CLHLS collects extensive data on demographics, socioeconomic conditions, psychological traits, health practices, and health conditions, and all data are collected via face-to-face interviews during in-home visits [37]. All respondents were tracked in the later waves unless death or loss to follow-up occurred. The response rates are 88 to 90% in the waves of CLHLS. Additional details, such as the sampling design, sampling weight and assessment of data quality, could be found in previous studies [38, 39].

**Fig. 1:**
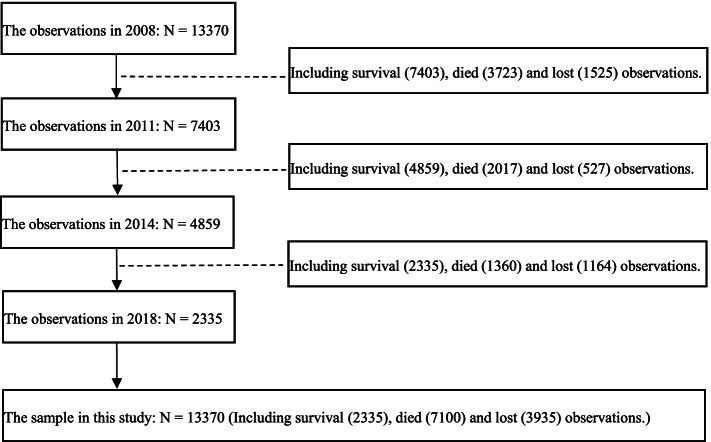
Flowchart describing sample selection

The data of this study were from the last four waves of datasets, including 2008, 2011, 2014 and 2018 waves. The total sample size of the four follow-up investigations was 16,954. Considering that there is insufficient information to confirm the age reported by extremely old people, this study excluded elderly individuals aged 105 and over [37, 40]. Moreover, participants who died in the following three months after the baseline interview were deleted, indicating that their death may be caused by other factors (e.g., accident, acute infectious disease), which cannot accurately measure the association between optimism and survival [41]. After excluding cases with missing information on key variables, 13,370 observations were retained in this study. By 2018, a total of 7100 elderly individuals died, 3935 elderly individuals were lost to follow-up, and 2335 elderly individuals were still alive. The flow diagram of study selection process is shown in Fig. 1.

### Measurements

#### Survival

The dependent variable in this study is the survival time of the interviewees during the whole observation period from 2008 to 2018. Information on death was obtained from the death questionnaire of CLHLS, and there were detailed death records. In the sample, the elderly who died were defined as sample of “event occurring”, lost follow-up or survived in 2018 were defined as sample of “truncation”. The death rate is 53.1% and the percentage of deletion is 46.9% in this study.

#### Optimism

Optimism was measured by Life Evaluation and Personality Scale (LEPS) of CLHLS. The LEPS was designed on the basis of the Life Orientation Test that is a classic scale to measure optimism [42]. The LEPS has been validated in the Chinese cultural context, showing acceptable internal consistency [43]. The 6 items, including “Do you always look on the bright side of things?” “Do you often feel fearful or anxious?” “Do you often feel lonely and isolated?” “Do you feel the older you get, the more useless you are, and have trouble doing anything?” “Dou you feel very happy with your life now?” and “Are you as happy as when you were younger?”. Respondents’ answers were provided on a 5-point Likert-type scale (1 = never to 5 = always). Some questions were asked about the opposite status, and we reverse-assigned the interview’s answer. Therefore, all items were summed to create a composite score that ranged from 6 to 30, with higher scores indicating higher levels of optimism. According to the cut-off value of optimism from previous literature, optimism was defined in 2 categories: optimistic (score > 21) and less optimistic (score ≤ 21) [27, 42, 44, 45]. In this study, older adults who were less optimistic will be used as a referent sample.

#### Health behaviors

Health behaviors were measured by 5 items similar to those in other studies [21, 30, 46], including physical activity, social activity, smoking status, and alcohol consumption. Physical activity included outdoor activities (Tai Ji, square dance, visiting friends, other outdoor activity) and garden work, which have 3 options: 1 = never, 2 = not every month but sometimes, 3 = almost every day. Social activity questions included “Do you now take part in social activities (organized) regularly?” Respondents’ answers were 1 = never, 2 = usually, and 3 = always. Smoking status and alcohol consumption were categorized as 1 = current, 2 = formerly and 3 = never. These 5 items were added to generate an overall health behaviors score ranging from 5 to 15, with higher scores indicating greater engagement in health promoting behaviors.

#### Covariates

Covariates included sociodemographic and family/social support, which were described in the previous literature [9, 21, 22]. Sociodemographic variables included age, gender (male vs. female), marital status (married vs. single/divorced/widowed), education level (no formal education (illiterate) vs. primary school, junior high school, senior high school, college or university graduate), residence (urban vs. rural), activities of daily living (ADL) and chronic diseases. ADL was the total score (ranging from 6 to 18) of six aspects of daily living abilities, including bathing, dressing, bathroom use, indoor transferring, continence and feeding. Depending on the independence of elderly individuals in completing each of the above actions, they were given a score of 1 = complete dependence on others, 2 = partial independence or 3 = complete independence, with a higher score indicating better daily living ability. Chronic diseases (range 0–30) were measured by the number of chronic diseases diagnosed by the doctor. The items consist of 30 different chronic diseases, such as hypertension and heart disease, with higher scores indicating a greater number of chronic diseases and worse health status. Family/social support variables included the elderly’s decision-making power in the family (1 = very low to 5 = very high), family economic status (1 = very poor to 5 = very rich), numbers of children (ranging from 0–13), co-residence with family members or not (1 = yes, 0 = no), medical insurance (1 = yes, 0 = no) and pension insurance (1 = yes, 0 = no).

#### Statistical analysis

We first reported descriptive statistics of the study sample. Internal consistency of optimism was verified with Cronbach’s alpha. Differences in covariates and health behaviors variables between participants who were optimistic and were less optimistic at baseline were tested by unpaired *t*-tests and chi-square tests for quantitative and categorical variables, respectively. Cox proportional hazard regression models were used to estimate the association of optimism and the rest of study variables with mortality.

Second, Cox proportional hazard models were used to estimate the association of optimism with mortality [47]. There are two assumptions in Cox proportional hazard model: proportional hazard and log-linearity. In our study, the outcome was survival, which was measured by the survival time and mortality of the interviewees during the whole observation period from 2008 to 2018. For the deceased individuals, time to death was calculated as the number of years from the baseline interview to the year of death. Participants who were not deceased and lost to follow-up due to reasons other than death were right-censored, and their survival time was measured as the years between the baseline and final recorded study year or last observation. The results are presented as adjusted hazard ratios (HRs) with 95% confidence intervals (CIs) for the analyses. Cox proportional hazards are applied to the survival to adjust for baseline group differences and provide a hazard ratio to quantify the effect that any single factor contributes to the survival, which has become the most widely applied regression perspective in survival analysis [48]. Based on the findings from the first set of analyses, we then investigated the effect of the association between optimism, health behaviors and mortality.

**Fig. 2:**
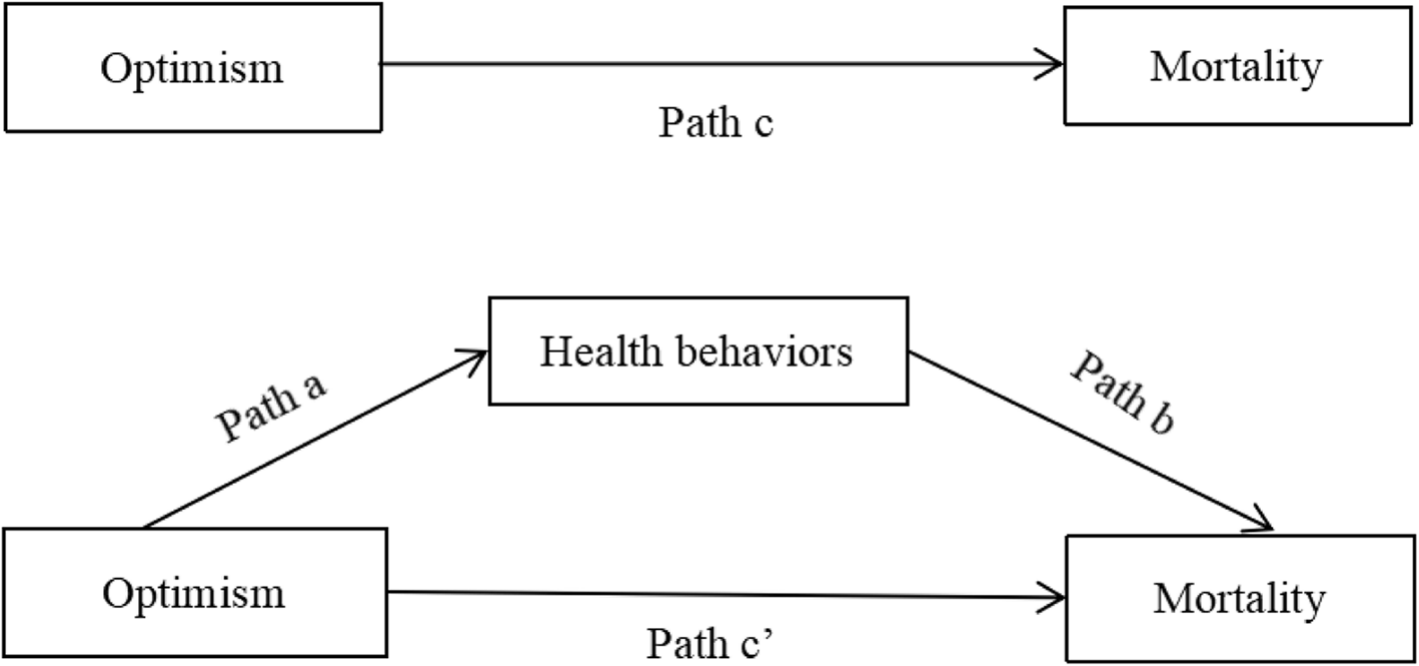
The mediation model proposed

Finally, we explored the mediating effect of health behaviors on the relationship between optimism and survival. Following the procedures proposed by Baron and Kenny, we adopted a three-step estimation for the mediator. The mediation effect analysis needs to meet the following conditions: (1) Optimism was significantly associated with mortality (Total effect; Path c, Fig. 2); (2) Optimism was significantly associated with health behaviors (Path a); (3) Controlling for optimism, health behaviors were significantly associated with mortality (Path b); (4) The relationship between optimism and mortality was reduced (Direct effect, Path c’) when controlling for health behaviors (Indirect effect, a × b) [49]. Recent studies found that the bootstrap confidence interval was among the most trustworthy tests when conducting a mediation analysis [50]. If the 95% bootstrap CIs does not contain 0, this particular indirect effect is considered statistically significant [51]. Statistical analyses were performed by using IBM SPSS Statistics version 24.

## Results

The Cronbach’s alpha coefficient was 0.77 for optimism, which suggested a moderate level of internal consistency. The elderly who was optimistic have a higher average score of health behaviors than the elderly who were less optimistic (13.41 vs. 13.27, *p* < 0.001). Participants who were optimistic were more likely than participants who were less optimistic to be male, younger, have a higher level of education, no spouse, a higher level of ADL, less kinds of chronic diseases, higher decision-making power in the family, higher level of family economic status, fewer children, health insurance, pension insurance, live in town and live with family members (Table 1).

**Table 1:** Descriptive statistics of analytic variables at baseline (*n* = 13,370)

Variable	Optimistic (*n* = 6821)		Less optimistic (*n* = 6549)		*P-*Value^#^
	**n**	**%**	**n**	**%**	
Gender					
Female	3343	49.01	3837	58.59	< 0.001
Male	3478	50.99	2712	41.41	
Education level					
No formal education (illiterate)	3498	51.28	4250	64.90	< 0.001
Primary school	1711	25.08	1455	22.22	
Junior high school	874	12.81	505	7.71	
Senior high school	407	5.97	186	2.84	
College or university graduate	331	4.85	153	2.34	
Marital status					
Married	3084	45.21	1930	29.47	< 0.001
Single/divorced/widowed	3737	54.79	4619	70.53	
Residence					
Town	3007	44.08	2411	36.81	< 0.001
Rural	3814	55.92	4138	63.19	
Co-residence with family members					
Yes	5852	85.79	5078	77.54	< 0.001
No	969	14.21	1471	22.46	
Medical insurance					
Yes	3722	54.57	2894	44.19	< 0.001
No	3099	45.43	3655	55.81	
Pension insurance					
Yes	1747	25.61	918	14.02	< 0.001
No	5074	74.39	5631	85.98	
	Mean	SD	Mean	SD	
Health behaviors	13.41	2.58	13.27	2.48	< 0.001
Age (years)	83.31	11.50	86.06	10.67	< 0.001
Chronic diseases	0.67	1.03	0.71	1.03	0.007
ADL	17.65	1.31	17.30	1.94	< 0.001
Decision-making power in the family	3.89	1.17	3.39	1.17	< 0.001
Family economic status	3.12	0.57	2.78	0.67	< 0.001
Numbers of children	4.30	2.12	4.36	2.22	0.065

*Abbreviations*: *ADL* activities of daily living, *SD* standard deviation

^**#**^*P-*values from *t*-tests (continuous variables) or chi-square tests (categorical variables)

**Fig. 3:**
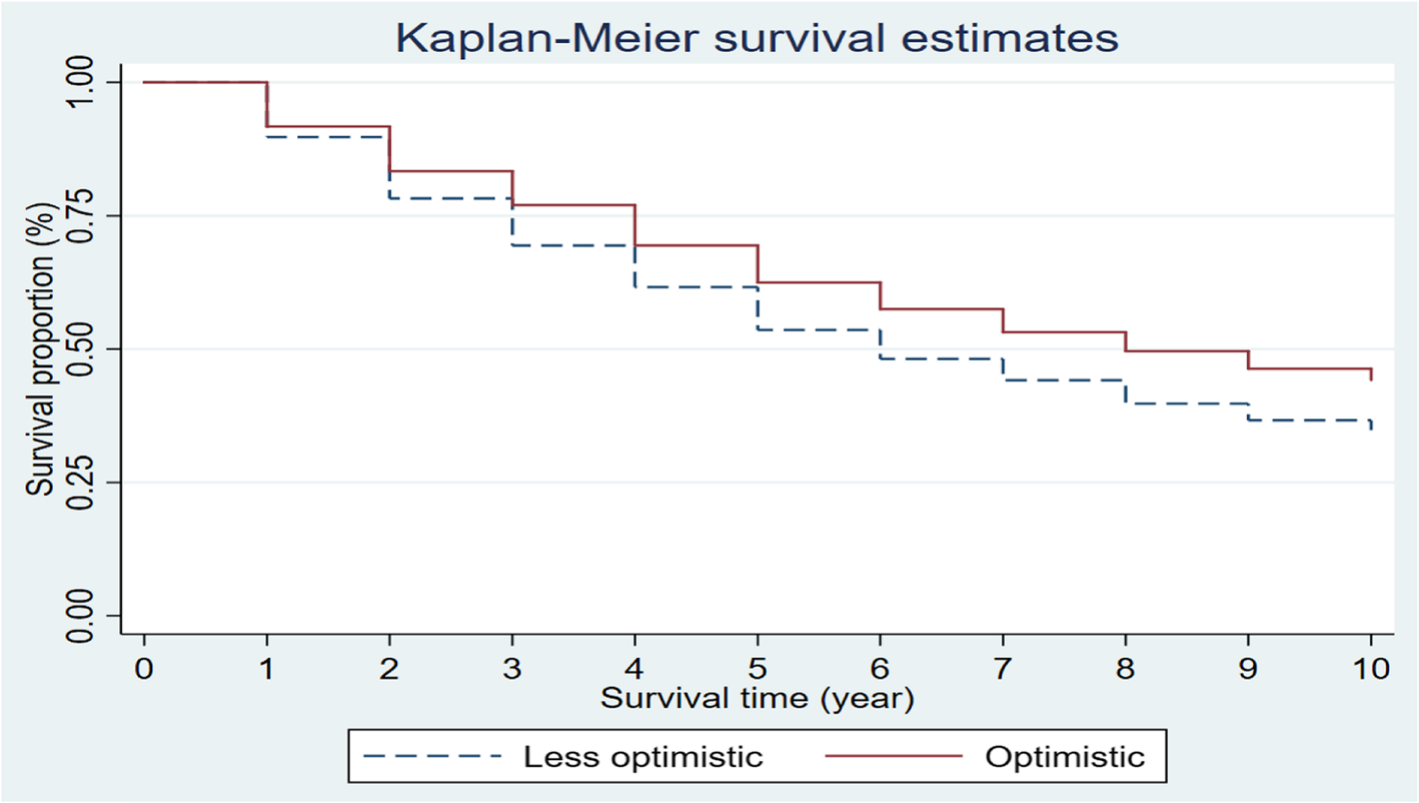
Kaplan–Meier analysis of survival according to two categories of optimism

To more intuitively show the relationship between optimism and the survival rate, we give the relationship graph between two categories of optimism and the survival rate. Figure 3 shows that the longer the follow-up time was, the lower the survival rate among the elderly. Compared to less optimistic individuals, optimistic individuals had a survival advantage (log rank test, *p* < 0.001). We found that optimism was positively associated with higher survival rates. According to Fig. 3, we could know that there may be a connection between optimism and survival rates, and we would further explore the relationship between optimism and mortality.

In Table 2, model 1 investigated mainly the association between sociodemographic and family/social support characteristics and mortality, and the outcomes showed that gender, age, marital status, residence, ADL, chronic diseases, decision-making power in the family, and co-residence with family members were significantly associated with elderly mortality. Model 2 investigated mainly the association between optimism and mortality after adjustment for sociodemographic and family/social support characteristics. Compared to the elderly who were less optimistic, optimistic older adults were significantly associated with a decreased mortality rate (HR = 0.93, 95% CI = 0.88 − 0.98). In model 3, health behaviors were included, and the protective effect of optimism on mortality decreased (HR = 0.94, 95% CI = 0.89 − 0.99), which showed that health behaviors are key elements that play a positive role in survival (HR = 0.95, 95% CI = 0.94 − 0.96), and optimism were more protective against survival than health behaviors.

**Table 2:** Hazard ratios of mortality by optimism over 10 years of follow-up

Variable	Model 1		Model 2		Model 3	
	**HR (95% CI)**	***p* value**	**HR (95% CI)**	***p* value**	**HR (95% CI)**	***p* value**
Optimism						
Less optimistic			1.00		1.00	
Optimistic			0.93 (0.89, 0.98)	0.008	0.94 (0.89 − 0.99)	0.014
Health behaviors					0.95 (0.94 − 0.96)	< 0.001
Age (years)	1.06 (1.05 − 1.06)	< 0.001	1.06 (1.05 − 1.06)	< 0.001	1.06 (1.05 − 1.06)	< 0.001
Gender						
Female			1.00		1.00	
Male	1.51 (1.43 − 1.60)	< 0.001	1.52 (1.43 − 1.61)	< 0.001	1.52 (1.44 − 1.61)	< 0.001
Education level						
No formal education (illiterate)	1.00		1.00		1.00	
Primary school	0.95 (0.89 − 1.02)	0.136	0.95 (0.89 − 1.02)	0.136	0.96 (0.90 − 1.03)	0.275
Junior high school	0.87 (0.79 − 0.96)	0.008	0.87 (0.79 − 0.97)	0.009	0.89 (0.80 − 0.98)	0.023
Senior high school	0.81 (0.70 − 0.94)	0.006	0.81 (0.70 − 0.94)	0.007	0.83 (0.72 − 0.97)	0.020
College or university graduate	0.73 (0.61 − 0.88)	< 0.001	0.73 (0.61 − 0.88)	< 0.001	0.76 (0.63 − 0.91)	0.002
Marital status						
Single/divorced/wid wed	1.00		1.00		1.00	
Married	0.85 (0.79 − 0.91)	< 0.001	0.85 (0.79 − 0.91)	< 0.001	0.85 (0.80 − 0.91)	< 0.001
Residence						
Rural	1.00		1.00		1.00	
Town	0.77 (0.73 − 0.81)	< 0.001	0.77 (0.73 − 0.82)	< 0.001	0.78 (0.74 − 0.82)	< 0.001
Co-residence with family members						
No	1.00		1.00		1.00	
Yes	1.09 (1.02 − 1.17)	0.011	1.10 (1.03 − 1.18)	0.006	1.10 (1.03 − 1.18)	0.004
Medical insurance						
No	1.00		1.00		1.00	
Yes	0.37 (0.35 − 0.39)	< 0.001	0.37 (0.35 − 0.39)	< 0.001	0.37 (0.35 − 0.39)	< 0.001
Pension insurance						
No	1.00		1.00		1.00	
Yes	0.86 (0.79 − 0.93)	< 0.001	0.87 (0.80 − 0.94)	< 0.001	0.90 (0.83 − 0.98)	0.010
ADL	0.96 (0.95 − 0.98)	< 0.001	0.96 (0.95 − 0.98)	< 0.001	0.97 (0.96 − 0.99)	< 0.001
Chronic diseases	1.03 (1.01 − 1.06)	0.021	1.03 (1.00 − 1.06)	0.032	1.03 (1.01 − 1.06)	0.015
Decision-making power in the family	0.98 (0.96 − 1.00)	0.040	0.98 (0.96 − 1.00)	0.116	0.99 (0.97 − 1.01)	0.397
Family economic status	0.98 (0.94 − 1.02)	0.347	0.99 (0.96 − 1.03)	0.765	1.00 (0.96 − 1.04)	0.835
Number of children	1.03 (1.02 − 1.04)	< 0.001	1.03 (1.02 − 1.04)	< 0.001	1.03 (1.02 − 1.04)	< 0.001

*Abbreviations: ADL* activities of daily living, *HR* hazard ratio, *CI* confidence interval

To further clarify the mechanism by which optimism is correlated with mortality, we examined the mediating effect of health behaviors on the relationship between optimism and mortality. The results are shown in Table 3. In Model 4, optimism was regressed on mortality, and optimism had a significant correlation with mortality (Path c: *β* = -0.070, 95% bootstrap CI = -0.12 − -0.02). In Model 5, optimism was regressed on health behaviors, and optimism had a positive correlation with health behaviors (Path a: *β* = 0.086, 95% bootstrap CI = 0.07 − 0.14). In Model 6, optimism and health behaviors were simultaneously regressed on mortality. The negative effect of optimism on mortality decreased (Path c’: *β* = -0.065, 95% bootstrap CI = -0.09 − -0.05), and health behaviors had a significant correlation with mortality (Path b: *β* = -0.053, 95% bootstrap CI = -0.09 − -0.05). The mediating effect was -0.005 (a × b = 0.086 × -0.053, 95% bootstrap CI = 0.03 − 0.09), and the 95% bootstrap CIs do not include zero, which showed that health behaviors played an intermediary role in the relationship between optimism and mortality. In addition, the total effect of optimism on mortality is -0.070, the direct effect is -0.065, and the indirect effect is -0.005. The results indicate that health behaviors partially mediated the effect of optimism on mortality, and that 7.15% of this effect can be explained by mediating effects.

**Table 3:** Analysis of mediating effect of health behaviors

	**Path**	***β***	**95% Bootstrap CI**	***p* value**	**Proportion of effect (%)**
Model 4 (Outcome: mortality)					
Optimism	c (Total effect)	-0.070	(-0.12 − -0.02)	0.008	100.00
Model 5 (Outcome: health behaviors)					
Optimism	a	0.086	(0.07 − 0.14)	0.030	–
Model 6 (Outcome: mortality)					
Optimism	c’ (Direct effect)	-0.065	(-0.09 − -0.05)	0.014	92.85
Health behaviors	b	-0.053	(-0.09 − -0.05)	< 0.001	–
Mediating effect	a x b (Indirect effect)	-0.005	(0.03 − 0.09)	–	7.15

*Note*: *CI* Confidence Interval. Model 4, optimism → mortality; Model 5, optimism → health behaviors; Model 6, optimism, health behaviors → mortality

^#^Overall test of association adjusted for gender, age, education level, marital status, residence, ADL, chronic diseases, decision-making power in the family, family economic status, number of children, co-residence with family members, medical insurance and pension insurance. The effects of covariates were omitted

## Discussion

The findings of this study highlight the importance of optimism and its positive influence on survival, and health behaviors played an intermediary role in the relationship between optimism and survival. Elderly individuals who were optimistic had a higher level of health behaviors and lower mortality than those who were less optimistic during 10 years of follow-up while controlling for sociodemographic and family/social support characteristics. Optimism was more protective against survival than health behaviors.

There are different explanations for how optimism affects survival among the elderly. One explanation is that optimism can reduce negative emotion (e.g., anxiety and stress) and reduce the incidence of various mental illnesses, which was discussed mainly from the perspective of psychological mechanisms. Optimism, as a key part of psychological resources, can help individuals perceive less stress by promoting positive relations with others, positive reappraisal and a problem-solving approach toward stressful situations, thereby reducing the activation of stress regulatory systems [15, 18]. Considering psychosocial pathways, more optimistic individuals may experience less extreme emotional reactivity to and faster recovery from acute stressors.Optimists think favorable outcomes are possible; they may be more likely to set goals and resolutely pursue goal-related tasks, persist in the face of challenge, seek information to deal with future health risks, act in ways that are consistent with reducing future dangers, and use effective coping strategies when they encounter stressors such as problem-focused coping [19, 52]. When faced with adversity, elderly individuals with more optimism show reduced physiological responses, including lower heart rate [21], blood pressure [11] and cortisol [26], which could obviously reduce the rate of mental illnesses [34] and mortality [9].

Another explanation is that optimismcould promote health behaviors to increase the chance of survival. The study found that elderly individuals with optimism and higher scores of health behaviors could promote their physical health. Optimism can directly increase engagement in health behaviors, which is consistent with the idea that optimism is positively associated with health behaviors independent of chronic illness, medication usage, and both individual and area-based indices of socio-economic status [3, 24, 25]. Theorists posit that individual with greater optimism enjoy better health outcomes because they engage in healthier behaviors such as exercising, eating fruits and vegetables, and avoiding cigarette smoking [2, 7, 26]. Healthier diet and physical activity are important elements for healthy aging, and optimism could increase the frequency of these health behaviors [27]. Another study found that optimism was a strong predictor of sleep quality, and older people who sleep better have healthier diets, more stable relationships and longer lives [53]. Optimism can improve the health literacy of the elderly, guide them to make health plans and avoid bad behavior habits, which plays an important role in improving the quality of life of the elderly [10].

The study found that health behaviors are a major predictor of mortality and positively influenced by optimism, and health behaviors mediate the association between optimism and survival. In our study, health behaviors were mainly composed of different levels of physical activity, social activity, smoking status and alcohol consumption, which played a key role in the health outcome and survival among older individuals. Moreover, health behaviors are often assessed with bipolar scales—for example, physical activity may be measured with a scale ranging from inactivity (i.e., sedentary behaviors) to vigorous activity. However, being inactive may have different correlates than vigorously engaging in exercise does (e.g., sedentary behaviors are associated with an increased risk of disease independent of physical activity) [33, 34]. In our study, sedentary behaviors were not included in the health behaviors, which ensured the consistency of unilateral measures of health behaviors. Smoking and alcohol consumption were key elements affecting mortality. Research has suggested that smoking intensity (i.e., years of smoking, number of cigarettes per day) influences associations between smoking and health outcomes [54]. Previous studies have found that optimism significantly reduces the frequency of smoking and alcohol consumption, which is an important factor in promoting their health [3, 55]. Therefore, smoking and consumption of alcohol were included in the health behaviors of our study, and empirical studies have illustrated that not smoking and low consumption of alcohol are significantly associated with reduced mortality among older adults.

Our study found that health behaviors reduced the mortality rate less than optimism. Health behaviors are an important mediator of the association between optimism and survival and are significantly associated with health outcomes and survival status. In fact, the mind often affects daily life through behaviors, and nourishing the mind and regulating behaviors have always been the pillars of health preservation advocated by traditional Chinese medicine [56]. Older people who have a negative emotion (e.g., pessimism) are more likely to be overeating and sedentary [57], but optimistic individuals are more likely to participate in social activities and maintain healthy eating habits [4]. How to maintain the consistency of mental status and daily actions and improve the levels of optimism and health behaviors are important tasks of health management for the elderly. However, we found that older adults did not receive high scores on health behaviors and the proportion of elderly individuals without optimism was high, which indicated that Chinese elderly adults are not in good mental and physical health. The Chinese government should further improve its public health policy, such as strengthening the construction of sports hardware facilities and promoting more health education and guidance. Interventions focused on health maintenance and improvement in elderly adults represent frequently combined health promotion and psychological disease prevention actions [58]. Only by strengthening their own health management can the elderly make positive psychology effectively guide health behaviors, to improve the quality of life in their aging process.

Several limitations should be considered. First, unmeasured confounding is always possible in observational studies. For example, a genetic predisposition to good health could lead to higher optimism. While we cannot completely rule out this possibility, we did control for a range of potential confounders, including ADL and chronic diseases. Additionally, strong genetic variants associated with optimism have not yet been found. Second, self-reported evaluation of health behaviors may be an imprecise method for estimating the type and duration of physical activity. In fact, physical activities consist of not only the frequency but also the time and intensity, all of which need to be measured. More precise measures of health behaviors should be involved in future studies. Furthermore, depression is a significant factor affecting mortality in the elderly. Generally, studies have included depression as an important control variable in the analysis of the relationship between optimism and mortality. However, variables of depression were not available in the CLHLS, which may introduce some biases.

## Conclusions

This study found that optimism and health behaviors are significantly associated with survival among elderly Chinese individuals. Optimism and health behaviors were positively correlated with survival, and the positive effect of optimism on survival was larger than the positive effect of health behaviors. Health behaviors played an intermediary role in the relationship between optimism and survival. Our findings suggest that optimism and health behaviors could promote health and longevity among the elderly. Policymakers should formulate valuable targets to promote individual health by fostering psychological resources and encouraging the elderly to engage in some kinds of health behaviors (e.g., exercising and eating more vegetables) and to participate in recreational and social activities, which could help them live longer in a healthier way. These findings have implications for understanding mental and behavioral factors that promote healthy and resilient aging.

## Data Availability

The datasets used are available from the corresponding author on reasonable request.

## Abbreviations

ADL: Activities of daily living
CI: Confidence interval
CLHLS: Chinese Longitudinal Health Longevity Survey
HR: Hazard ratio
LEPS: Life Evaluation and Personal Scale
SD: Standard deviation
